# Complete Chromosome Sequence of *Carnobacterium maltaromaticum* LMA 28

**DOI:** 10.1128/genomeA.00115-12

**Published:** 2013-01-31

**Authors:** Catherine Cailliez-Grimal, Stéphane Chaillou, Jamila Anba-Mondoloni, Valentin Loux, Muhammad Inam Afzal, Abdur Rahman, Gilles Kergourlay, Marie-Christine Champomier-Vergès, Monique Zagorec, Paw Dalgaard, Jorgen J. Leisner, Hervé Prévost, Anne-Marie Revol-Junelles, Frédéric Borges

**Affiliations:** a Université de Lorraine, Laboratoire d’Ingénierie des Biomolécules (LIBio), Vandoeuvre-lès-Nancy, France; b INRA, UMR1319 Micalis, Jouy-en-Josas, France; c AgroParisTech, UMR Micalis, Jouy-en-Josas, France; d Unité de Mathématique, Informatique et Génome, INRA, Jouy-en-Josas, France; e INRA, Nantes, France; f L’Université Nantes Angers Le Mans (L’UNAM), Oniris, UMR 1014 Secalim, Nantes, France; g National Food Institute (DTU Food), Technical University of Denmark, Kgs. Lyngby, Denmark; h Department of Veterinary Disease Biology, Faculty of Health and Medical Sciences, University of Copenhagen, Copenhagen, Denmark

## Abstract

Within the lactic acid bacterium genus *Carnobacterium, Carnobacterium maltaromaticum* is one of the most frequently isolated species from natural environments and food. It potentially plays a major role in food product biopreservation. We report here on the 3.649-Mb chromosome sequence of *C. maltaromaticum* LMA 28, which was isolated from ripened soft cheese.

## GENOME ANNOUNCEMENT

The genus *Carnobacterium* belongs to the lactic acid bacteria, and currently consists of 11 species. *Carnobacterium maltaromaticum* strains are widely found in foods, including dairy products (1). This species has potential for application as a protective culture in foods. Most research has focused on the production of bacteriocins, on their roles in the inhibition of *Listeria monocytogenes*, and on the regulation of metabolic pathways of sensory importance (2). *C. maltaromaticum* LMA 28 was isolated from a soft ripened cheese (3).

The genome sequence of *C. maltaromaticum* LMA 28 was determined using 454 pyrosequencing GS-FLX system (Roche 454 Life Sciences, Mannheim, Germany) and Illumina sequencing. Pyrosequencing runs, including shotgun and paired-end runs, resulted in 15 scaffolds containing 123 contigs and 55-fold coverage. A subsequent Illumina sequencing run performed with a paired-end library corrected 923 indels. PCR-base techniques and Sanger sequencing of the products were used to close the remaining gaps. The manually curated sequence of LMA 28 comprises one chromosome of 3,649,737 bp with an overall G+C content of 34.5%. Coding sequence (CDS) predictions and annotations were performed with Integrative Services for Genomics Analysis (ISGA) (4) and provided 3,933 predicted CDSs, 59 tRNA genes, 6 rRNA operons, and a single 5S rRNA gene.

So far, three genomic sequences of *Carnobacterium* have been published: the complete genome sequence of *Carnobacterium* sp. 17-4 (5) isolated from permanent cold seawater; the draft genome sequences of *C. maltaromaticum* ATCC 35586, isolated from a diseased salmon (6); and the draft genome of *Carnobacterium* sp. AT7, a piezophilic strain isolated from the Aleutian trench (7). The genome size of the strain ATCC 35586 (3.5 Mbp) is similar to that of LMA 28, and both are approximately 1 Mbp larger than the genomes of *Carnobacterium* sp. 17-4 and AT7 (2.6 Mbp and 2.4 Mbp, respectively). The larger chromosomal size of *C. maltaromaticum* might explain the ability of this species to adapt to multiple and diverse environments compared to the other carnobacterial species. This genomic trait is illustrated by the presence of genes involved in the metabolism of branched-chain amino acids.

Indeed, the species *C. maltaromaticum* is well known for its ability to produce the flavor compound 3-methylbutanal, which is the result of leucine catabolism. In lactic acid bacteria, the more prevalent pathway is the α–keto acid dehydrogenase (KaDH) pathway (8). A less common alternative pathway is the α–keto acid decarboxylase pathway, encoded by gene *kdcA*. This gene is only described for two strains of *L. lactis* (8). In the genome sequences of *C. maltaromaticum* LMA 28 and ATCC 35586, an orthologous gene of *kdcA* was found (2) that is absent from the genomes of the other *Carnobacterium* strains.

### Nucleotide sequence accession number.

The complete chromosome sequence of *Carnobacterium maltaromaticum* LMA 28 has been deposited at EMBL/GenBank under the accession number no. HE999757.

## References

[1] Afzal MI Jacquet T Delaunay S Borges F Millière JB Revol-Junelles AM Cailliez-Grimal C 2010 Carnobacterium maltaromaticum: identification, isolation tools, ecology and technological aspects in dairy products. Food Microbiol. 27:573–579 20510773 10.1016/j.fm.2010.03.019

[2] Leisner JJ Laursen BG Prévost H Drider D Dalgaard P 2007 *Carnobacterium*: Positive and negative effects in the environment and in foods. FEMS Microbiol. Rev. 31:592–613 17696886 10.1111/j.1574-6976.2007.00080.x PMC2040187

[3] Millière JB Michel M Mathieu F Lefebvre G 1994 Presence of *Carnobacterium* spp. in French surface mould-ripened soft-cheese. J. Appl. Bacteriol. 76(3):264–269

[4] Hemmerich C Buechlein A Podicheti R Revanna KV Dong Q 2010 An Ergatis-based prokaryotic genome annotation web server. Bioinformatics 26:1122–1124 20194626 10.1093/bioinformatics/btq090

[5] Voget S Klippel B Daniel R Antranikian G 2011 Complete genome sequence of *Carnobacterium* sp. 17-4. J. Bacteriol. 193:3403–3404 21551290 10.1128/JB.05113-11 PMC3133254

[6] Leisner JJ Hansen MA Larsen MH Hansen L Ingmer H Sørensen SJ 2012 The genome sequence of the lactic acid bacterium, *Carnobacterium maltaromaticum* ATCC 35586 encodes potential virulence factors. Int. J. Food Microbiol. 152:107–115 21704418 10.1016/j.ijfoodmicro.2011.05.012

[7] Lauro FM Chastain RA Blankenship LE Yayanos AA Bartlett DH 2007 The unique 16S rRNA genes of piezophiles reflect both phylogeny and adaptation. Appl. Environ. Microbiol. 73:838–845 17158629 10.1128/AEM.01726-06 PMC1800765

[8] Liu M Nauta A Francke C Siezen RJ 2008 Comparative genomics of enzymes in flavor-forming pathways from amino acids in lactic acid bacteria. Appl. Environ. Microbiol. 74:4590–4600 18539796 10.1128/AEM.00150-08 PMC2519355

